# Sex in murky waters: algal-induced turbidity increases sexual selection in pipefish

**DOI:** 10.1007/s00265-017-2310-8

**Published:** 2017-04-05

**Authors:** Josefin Sundin, Tonje Aronsen, Gunilla Rosenqvist, Anders Berglund

**Affiliations:** 1 Department of Ecology and Genetics/Animal Ecology, Uppsala University, Uppsala, Sweden; 2 Department of Neuroscience, Uppsala University, Uppsala, Sweden; 3 Centre for Biodiversity Dynamics, Department of Biology, Norwegian University of Science and Technology, Trondheim, Norway; 4 Norwegian Institute for Nature Research (NINA), Trondheim, Norway

**Keywords:** Climate change, Eutrophication, Syngnathidae, Baltic Sea, Sex-role reversal

## Abstract

**Abstract:**

Algal-induced turbidity has been shown to alter several important aspects of reproduction and sexual selection. However, while turbidity has been shown to negatively affect reproduction and sexually selected traits in some species, it may instead enhance reproductive success in others, implying that the impact of eutrophication is far more complex than originally believed. In this study, we aimed to provide more insight into these inconsistent findings. We used molecular tools to investigate the impact of algal turbidity on reproductive success and sexual selection on males in controlled laboratory experiments, allowing mate choice, mating competition, and mate encounter rates to affect reproduction. As study species, we used the broad-nosed pipefish, *Syngnathus typhle*, a species practicing male pregnancy and where we have previously shown that male mate choice is impaired by turbidity. Here, turbidity instead enhanced sexual selection on male size and mating success as well as reproductive success. Effects from mating competition and mate encounter rates may thus override effects from mate choice based on visual cues, producing an overall stronger sexual selection in turbid waters. Hence, seemingly inconsistent effects of turbidity on sexual selection may depend on which mechanisms of sexual selection that have been under study.

**Significance statement:**

Algal blooms are becoming increasingly more common due to eutrophication of freshwater and marine environments. The high density of algae lowers water transparency and reduces the possibility for fish and other aquatic animals to perform behaviors dependent on vision. We have previously shown that pipefish are unable to select the best partner in mate choice trials when water transparency was reduced. However, fish might use other senses than vision to compensate for the reduction in water transparency. In this study, we found that when fish were allowed to freely interact, thereby allowing competition between partners and direct contact between the fish, the best partner was indeed chosen. Hence, the negative effects of reduced water visibility due to algal blooms may be counteracted by the use of other senses in fish.

**Electronic supplementary material:**

The online version of this article (doi:10.1007/s00265-017-2310-8) contains supplementary material, which is available to authorized users.

## Introduction

Human-induced changes, such as climate change (Bradshaw and Holzapfel 2006), pollution (Carson 1962; Lurling and Scheffer 2007), selective harvesting (Law 2000; Coltman et al. 2003), urbanization (Marzluff et al. 2008), deforestation, and habitat fragmentation (Saunders et al. 1991; Skole and Tucker 1993), affect the abundance, distribution, and evolution of organisms. An organism’s resilience to such fast and drastic environmental change largely depends on its ability to disperse, its mobility, its behavioral plasticity, and the fitness consequences related to the environmental change (Dall et al. 2005; Donaldson-Matasci et al. 2008). The severity and speed of changes induced by humans imply that organisms need to adapt to the new environment at a very short time scale in order to persist (Tuomainen and Candolin 2010; Sih 2013; Wong and Candolin 2015).

Coastal ecosystems are particularly sensitive to human-induced environmental change such as eutrophication, which can lead to an extreme growth of phytoplankton and filamentous algae (Smith 2003). Algal-induced turbidity decreases water transparency, narrows the light spectrum (Jerlov 1976; Seehausen and van Alphen 1998), and changes chemical properties of the water (Lapointe and Matzie 1996; Perus and Bonsdorff 2004). Species vary greatly in how they respond to turbidity. For example, turbidity and increased algal cover may hamper species-specific sexual signaling, leading to hybridization in Lake Victoria cichlids (Seehausen et al. 1997; Selz et al. 2014). It can relax sexual selection on male nuptial coloration and courtship activity, diminishing honest signaling of mate quality and increasing the probability of reproduction for parasitized males, as shown in the three-spined stickleback (Candolin et al. 2007; Wong et al. 2007; Heuschele and Candolin 2010). Turbidity can further relax sexual selection on mate size and decrease parental care in the sand goby (Järvenpää and Lindström 2004, 2011), increase latency until courting in the desert goby (Michelangeli et al. 2015), and decrease mate search and evaluation in the sailfin molly and in the three-spined stickleback (Heubel and Schlupp 2006; Heuschele et al. 2012). All these studies thus show adverse effects of turbidity in traits and behaviors relevant for population viability. By contrast, turbidity and increased algal cover have been shown to enhance reproductive success through increased reproductive lifespan and increased hatching success in the three-spined stickleback (Candolin et al. 2008, 2014) and increased egg survival in the sand goby (Järvenpää and Lindström 2011). These conflicting results imply that the impact of eutrophication is far more complex than originally believed, in particular when studied under more natural conditions. The object of this study is therefore to increase our understanding on the effects of turbidity on sexual selection under more natural conditions by exploring additional mechanisms of sexual selection, such as mating competition and mate encounter rates.

We experimentally investigated the impact of algal turbidity on mating probability (proportion of mated males), mating success (number of partners), and reproductive success (number of eggs), using the broad-nosed pipefish, *Syngnathus typhle*. Turbidity may have particularly severe consequences for *S. typhle* since this species relies on visual cues in mate choice (Rosenqvist and Berglund 2011). Furthermore, this pipefish is probably not able to completely compensate for a reduction in visibility by switching to other sensory systems since chemoreception (Sundin et al. 2010; Lindqvist et al. 2011) as well as sound (Ripley and Foran 2007, investigated for *S. floridae* and *S. fuscus*) seem less important in mate search or mate choice for this fish. However, chemoreception should not be completely ruled out due to the use of MHC during mate choice in this species (Roth et al. 2014). Both males and females are choosy, with body size being an important mate choice cue (Berglund et al. 1986b; Sandvik et al. 2000). Fecundity increases with size (Berglund et al. 1986a, b; Sandvik et al. 2000), which is true for many other species across a broad range of taxa, and mating preference for large body size is thus very common (Andersson 1994). We have previously shown that sexual selection on mate size through mate choice of visual cues is impaired in turbid waters (Sundin et al. 2010). We now set out to explore how additional mechanisms of sexual selection, such as mating competition and mate encounter rates, affect sexual selection on mate size in turbid waters. We did this by allowing males and females to interact freely in controlled mating experiments in clear or turbid water. We subsequently assessed the genetic mating system using microsatellite markers. We used fish from the Baltic Sea population which has experienced high levels of turbidity (Bonsdorff et al. 2002; Salonen et al. 2009; Veneranta et al. 2011) and may thus be adapted to these conditions. This is suggested by its overall robustness to hypoxic conditions (which may be caused by algal blooms) during a mating event (Sundin et al. 2015) and the lack of a turbidity effect on the development of sexual ornaments in the closely related straight-nosed pipefish, *Nerophis ophidion* (Sundin et al. 2016).

Given the findings in the majority of previous studies on effects of turbidity on sexual selection cited above, we still hypothesized that turbidity would relax sexual selection on mate size and decrease mating as well as reproductive success.

## Methods

### Catching and handling

We collected pipefish in the Baltic Sea, at Kyllaj on the east coast of Gotland, Sweden (57° 44′ N, E18° 57′ E) in May 2010. We trawled shallow (<10 m deep) meadows of eelgrass, *Zostera marina*, using a beam trawl (mesh size 4 mm) pulled by a motorboat. In the laboratory at Ar research station, we kept the pipefish separated by sex in 650-l holding tanks (12 tanks in total) with continuously renewed seawater and artificial eelgrass for shelter. Temperature and salinity followed local conditions during the holding period (holding period May–June: temperature 8–20 °C, salinity 6.0–6.5‰). The light cycle was set to L17h/D7h. We fed the fish three times a day with live and frozen mysid shrimps and laboratory raised *Artemia* sp. Tanks were cleaned daily. The same feeding regime was also applied during the experiment.

### Experimental design

The experiment investigating the impact of algal turbidity on mating probability (proportion of mated males), mating success (number of partners), and reproductive success (number of eggs) ran from June 23 to 30, 2010. We allowed four females and four males to freely interact and breed in either clear or turbid water (16 replicates per treatment) during 4 days (which is ample time for the fish to mate; Berglund et al. 1989; Jones et al. 2005). Prior to the experiment, we measured standard length of all fish, and for females, we also measured body depth. Fish length and depth overlapped between treatments (mean ± SD, *N* = 64 fish per sex and treatment (apart from female turbid where *N* = 63, since one female turned out to be a male), length: females: clear 135.1 ± 13.7 mm, turbid 137.5 ± 12.7 mm, males: clear 132.1 ± 9.6 mm, turbid 133.5 ± 10.9 mm; female depth: clear 6.5 ± 1.1 mm, turbid 6.6 ± 1.1 mm). We also looked for skin parasites (*Cryptocotyle* sp.) as parasites can make mates unattractive (Rosenqvist and Johansson 1995). If larger fish had more parasites than smaller fish, this could have induced complications for measurements of sexual selection on mate size. No fish included in the final analyses had more than four visible *Cryptocotyle* sp., and there was no correlation between fish size and number of parasites (linear regression: *r* ^2^ = 0.03, *F*_1,96_ = 2.52, *P* = 0.116,). For breeding tanks, we used 650-l tanks (1 × 1 × 1 m, water depth 0.65 m), thus generating a density of 0.01 fish per liter water (8 fish in 650 l), which allows sexual selection to operate (Aronsen et al. 2013a, b). The tanks were equipped with artificial eelgrass for shelter and air stones for oxygen supply. For the turbid treatment, we manipulated algal turbidity by adding frozen *Nannochloropsis* sp. algae (Instant Algae Nanno 3600®, Microalgae), mixed with seawater prior the start of the experiment, to reach the targeted turbidity level of 10 nephelometric turbidity units (NTU). This corresponds to high, but not extreme, turbidity levels in the Baltic Sea (Salonen et al. 2009; Veneranta et al. 2011). The aeration of the water prevented algae from sinking to the bottom and kept the turbidity level relatively constant. Turbidity and temperature were measured two to three times per day using a HANNAH HI93703 turbidity meter. There were no differences in temperature between treatments (mean ± SD, 136 measurements per treatment: clear 16.7 ± 1.9 °C, turbid 16.8 ± 2.0 °C). Mean turbidity levels between replicates in the turbid treatment (144 measurements in total) varied from 9.43 to 10.69 NTU, overall turbidity varied between 8.16 and 13.16 NTU (mean 10.07 ± 0.9). Turbidity levels of clear water were below the threshold level of the turbidity meter (0.5 NTU).

On day 4 (the last day of each set of replicates), we fin-clipped the females by cutting approximately 3 mm^2^ of tissue from the caudal fin, after which females were released back into the wild at approximately the site of capture, along with non-pregnant males. Pregnant males were transferred to 21-l flow-through brooding aquaria equipped with artificial eelgrass for shelter, each replicate separate. We kept the males until the embryos had developed eyespots to enable parentage analysis of the embryos (Jones et al. 1999). We then euthanized the males by giving them an overdose of the sedative 2-phenoxyethanol (2 ml/l), after which we severed their spinal column above the operculum. Males were then stored in 95% ethanol, with embryos kept in the pouch of the males. It was not possible to record data blind due to the turbidity treatment, which is visible to any observer.

### Parentage analysis

To investigate the impact of algal turbidity on mating success and reproductive success, we assessed parentage using three highly polymorphic microsatellite loci (*Typh*04, *Typh*16, and *Typh*18), developed by Jones et al. (1999). All eggs were dissected from the membrane of the father’s brood pouch using flame-sterilized forceps, mapping their position within the brood pouch as they were extracted. Also, dead/unfertilized eggs were dissected and their position noted. Since eggs are spatially clumped by maternity in the male brood pouch, genotyping every fourth embryo gives enough resolution to assign the eggs to each mother (Jones and Avise 1997; Jones et al. 1999). The methods for DNA extraction, amplification of microsatellite loci, and scoring of fragment sizes are described in Aronsen et al. (2013a).

Mothers were assigned to each offspring using Cervus 3.0 (Kalinowski et al. 2007). Cervus assigned 99% of the offspring to a candidate mother with 95% confidence. The remaining 1% was assigned to a mother manually by using the information from the genotyped embryos located before and after the unassigned embryo. When the assigned embryos before and after the unassigned embryo belonged to different mothers, we assigned half of the embryos in-between (i.e., two embryos, as every fourth embryo was genotyped) to each of the mothers.

### Males affected by mold

During the pregnancy, males are very sensitive to all forms of infections (Landis et al. 2012; Sagebakken et al. 2016). Unfortunately, during the brooding period, many males got an infection most likely caused by *Saprolegnia*. Water molds (oomycetes) of the genus *Saprolegnia* cause Saprolegniosis, which is a disease that is characterized by visible white or gray patches of filamentous mycelium on the body or fins of fishes (van West 2006). Out of the 49 mated males, 26 were infected in the turbid treatment, and 11 out of 49 in the clear water treatment, meaning that more males were infected in the turbid than the clear treatment (GLM (using binomial response variable, 1 = infected, 0 = not infected): z-value = 2.86, *P* = 0.004, intercept (clear treatment): estimate ± SE (logit scale) = −1.57 ± 0.33, treatment (turbid): estimate ± SE (logit scale) = 1.19 ± 0.42, estimate for the turbid treatment is given as the contrast to the intercept (clear treatment)). The embryos carried by the infected fish could not be included in the parentage analyses. However, in neither the turbid nor the clear treatment did the males with and without the mold infection differ significantly in length (turbid: mean ± SD: infected 137.0 ± 8.64 mm, uninfected 135.9 ± 8.40 mm, Wilcoxon signed-rank: *W* = 0.365, *P* = 0.08, clear: mean ± SD: infected 127.5 ± 10.24 mm, uninfected 133.5 ± 8.68 mm, Wilcoxon signed rank: *W* = 380, *P* = 0.12). Hence, while the unfortunate mold infection caused a decrease in sample size, it did not cause a skew in size of the fish available for parentage analyses and should thus not influence the measures of sexual selection.

### Statistical analysis

To investigate whether algal turbidity had an impact on mate choice and mating success, we tested if there was any effect of treatment on proportion of mated fish and on mating (number of partners) or reproductive (number of eggs) success. For the models of proportion of mated fish, mating success, and reproductive success, we included replicate as a random effect (since fish within a replicate are not independent from each other), while treatment was the fixed factor (with two levels: clear and turbid). Length was included as a covariate. We used the method of Zuur et al. (2009) for model selection. We removed non-significant interactions (*P* > 0.1) from the final model using log likelihood tests (LRT) based on maximum likelihood. Only the minimum adequate models are presented (Table 1).

**Table 1:** The effect of treatment (clear and turbid) and length (mm) on male mating probability (proportion of mated males), mating success (number of partners), and reproductive success (number of eggs)

Response		Estimate (±SE)	*z*	*P*
Mating probability	Intercept (clear)	0.73 (4.07)	0.18	0.858
	Treatment (turbid)	−17.17 (6.61)	−2.60	0.009
	Length	0.003 (0.03)	0.11	0.911
	Treatment (turbid)*length	0.13 (0.05)	2.59	0.010
Mating success	Intercept (clear)	−1.11 (1.71)	−0.65	0.514
	Treatment (turbid)	−6.41 (2.89)	−2.22	0.027
	Length	0.01 (0.01)	0.87	0.385
	Treatment (turbid)*length	0.04 (0.02)	2.12	0.034
Reproductive success	Intercept (clear)	0.04 (5.08)	−0.03	0.978
	Treatment (turbid)	−18.36 (7.53)	−2.44	0.015
	Length	0.02 (0.04)	0.43	0.671
	Treatment (turbid)*length	0.13 (0.06)	2.35	0.019

All tests are GLMMs with replicate as a random factor. Estimates are reported as contrasts to the intercept

For the analysis of proportion of mated fish, we used a generalized linear mixed effect model (GLMM), with binominal error structure and logit-link function. Mated or not mated was the response variable (*n* = 64 males per treatment).

For the analysis of mating success (data gained from the parentage analysis), we used a GLMM with a poisson error distribution and log-link function. Number of partners (0–4) was the response variable (*n* = clear 53 males (16 replicates), turbid 38 males (16 replicates), i.e., including all mated males that were not infected and males that had not mated (0 partners) from all 16 replicates per treatment). Remaining males could not be included since the parentage analysis could not be conducted as the males had undeveloped eggs or had gotten a mold infection during the pregnancy.

The analysis of reproductive success (data gained from the parentage analysis) was equivalent to the analysis of mating success, with the exception that an individual level random effect was added to the model to account for overdispersion. The number of developed embryos was the response variable for males (since we genotyped every fourth embryo, we calculated reproductive success as four times the number of embryos that showed signs of embryo-development and hence could be genotyped). Males excluded in the prior analysis were again excluded for the same reasons.

Females were not analyzed due to the infection in many males (i.e., rendering data on female mating and reproductive success unreliable as we cannot know, with any certainty, which females that contributed eggs to the infected males), as described above. All analyses were performed using R version 2.13.1 (R Development Core Team 2011), with the Ime4 package (Bates et al. 2011).

## Results

### Mating probability

**Fig. 1:**
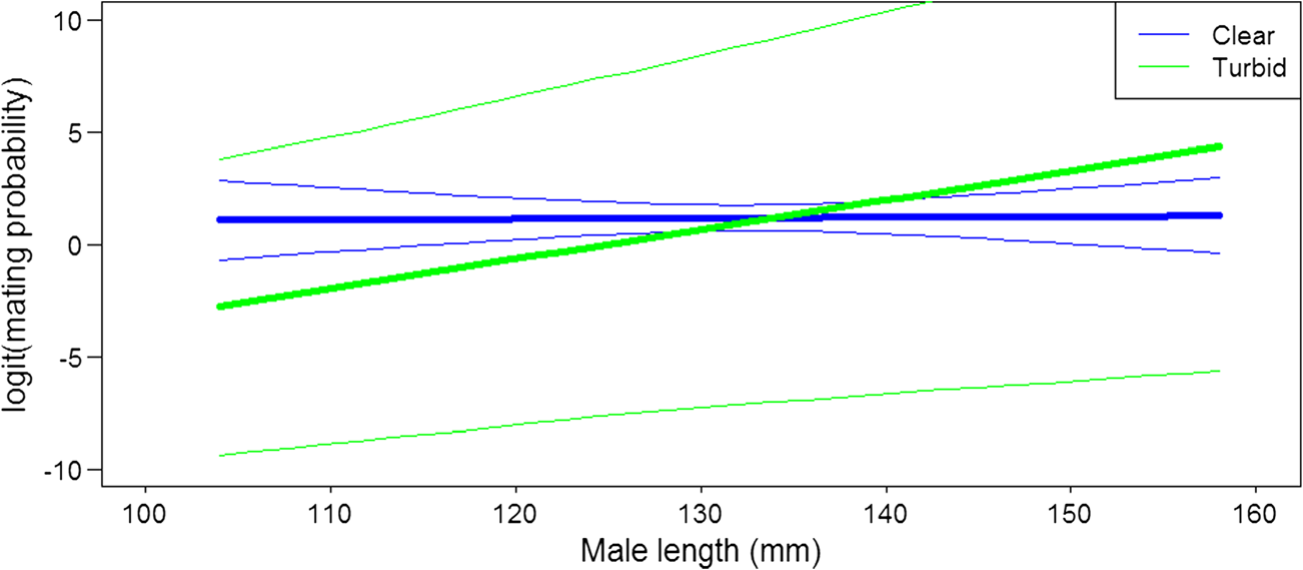
Estimated relationships from GLMM between mating probability (logit scale) and male length in the clear (*blue line*) and turbid (*green line*) treatment. *Thin lines* indicate 95% confidence intervals and have the same line color (*blue and green*) as the treatment they belong to. Estimates of the probability of mating are from the model presented in Table 1

Out of the 64 males, 49 mated in the turbid as well as in the clear treatment. The interaction between male body length and turbidity was significant (Fig. 1, Table 1), and when the relationship between mating probability and male body length was tested separately for the two treatments, there was a significant positive relationship between male length and mating probability in the turbid treatment (GLMM: estimated slope ± SE (logit scale) = 0.16 ± 0.06, z-value = 2.84, *P* = 0.005). In contrast, there was no relationship between male length and the mating probability in the clear treatment (GLMM: estimated slope ± SE (logit scale) = 0.003 ± 0.03, z-value = 0.11, *P* = 0.91) (Fig. 1, Table 1).

### Mating success

**Fig. 2:**
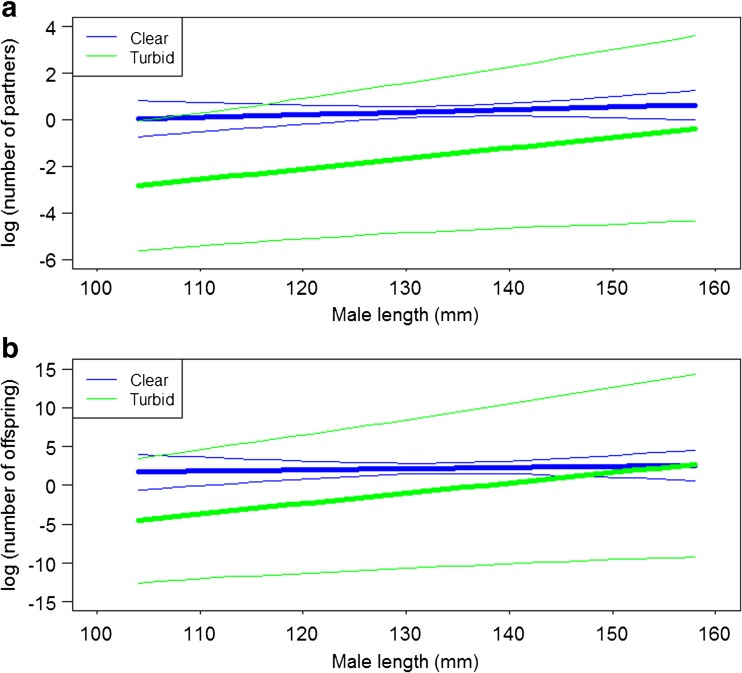
Estimated relationships (logarithmic scale) from GLMMs between the number of partners (**a**) and number of offspring (**b**) and male length in the clear (*blue line*) and turbid (*green line*) treatment. *Thin lines* indicate 95% confidence intervals and have the same line color (*blue and green*) as the treatment they correspond to. The estimated relationships are from the models presented in Table 1

Larger males mated with more females, and the relationship between male body length and number of partners was stronger in turbid environments (Fig. 2a, Table 1). The difference in the relationship between male length and number of partners in the turbid and clear treatments was due to small males having more partners in the clear treatment than in the turbid treatment. The median male length was 143 mm; we therefore tested for differences in mating success between treatments for males less than 143 mm (small males) or males that were 143 mm or longer (large males). Males less than 143 mm in length had significantly more mates in the clear than in the turbid treatment (Wilcoxon rank sum test: *W* = 863.5, *P* = 0.04). There were, however, no differences in the number of partners between males from the turbid and clear treatment when only considering males that were 143 mm or longer (Wilcoxon rank sum test: *W* = 23.5, *P* = 0.71).

### Reproductive success

Reproductive success followed the same pattern as mating probability and mating success, with a significant interaction between length and treatment, showing that the positive relationship between male body length and number of eggs received was stronger in the turbid treatment (Fig. 2b, Table 1).

## Discussion

The available studies on the effects of turbidity on sexual selection show very mixed results, sometimes demonstrating an increase, sometimes a decrease, and sometimes no effect on sexual selection (Järvenpää and Lindström 2004, 2011; Candolin et al. 2007, 2014; Wong et al. 2007; Heuschele and Candolin 2010). Here, we provide more insight into these inconsistencies: in a previous experiment, when we allowed mate choice on visual and/or chemosensory cues to influence the outcome, turbidity had negative effects on sexual selection and the addition of chemosensory cues could not counteract the reduction in visibility (Sundin et al. 2010). When allowing full-contact between the sexes, i.e., mating competition and mate encounter rates in addition to visual cues, as in this study, mimicking more natural conditions, things changed dramatically: turbidity actually increased sexual selection on body size in males. Most likely, the effects from mating competition and/or altered mate encounter rates under turbidity overrode possible effects from a hampered mate choice, producing a net increase in sexual selection on male length. The increased selection on male length was due to a reduction in mating and reproductive success in small males (Fig. 2a, b). This may be due to two reasons: small males did worse in mating competition under turbidity or they suffered a decrease in mate encounter rates. The former seems less likely, as male-male competition is not overwhelmingly strong in this sex-role reversed species (Berglund and Rosenqvist 2003). The possibility that small individuals are less frequently encountered or not correctly identified as potential mates under turbid conditions may, however, be more likely: random encounters should be more frequent with larger individuals, or larger individuals are more easily identified as potential partners than are small individuals under low visibility regimes. Turbidity in itself does not seem to have an effect on overall swimming activity, as shown in *S. typhle* (Sundin et al. 2010) as well as in the closely related straight-nosed pipefish, *Nerophis ophidion* (Sundin et al. 2016). Unfortunately, we could not assess sexual selection in females due to the mold outbreak.

The mold outbreak affected many pregnant males when we reared them for maternity analysis, and the males from the turbidity treatment were worse infected than males from the clear treatment. The infected males could still be used for assessing mating probability, but not for assessing mating or reproductive success. In the latter two analyses, we consequently overestimate the proportion of unmated males by including replicates with many infected males, as only mated males were affected. Since males from the turbidity treatment were worse affected than the clear water treatment, this could have skewed our results, but pregnant males from the turbid treatment without the infection did not differ from pregnant males with the infection in body size. Hence, the size-specific results that we found here were most likely not confounded by the mold infection, since the infection was size indiscriminate. Hence, we believe that the result that sexual selection acted stronger in turbid water by decreasing mating and reproductive success in small males still holds. The fact that the males from the turbid treatment were infected to a greater extent than males from the clear treatment is still an interesting finding in itself, and it suggests that those males for unknown reasons were more susceptible to the mold infection. This study was not designed to investigate the possible effects of turbidity on infection susceptibility, and to our knowledge, this has not been explicitly tested in fish. Sirois and Dodson (2000) found that larval rainbow smelt (*Osmerus mordax*) from a collection site with high turbidity (50 NTU) had a higher prevalence of cestode parasites compared to fish collected from a site with lower turbidity (20 NTU). Although there were additional differences between the two collection sites, their finding follows our observation that high turbidity may lead to higher susceptibility for infection, and it offers an interesting venue for further research.

Both the probability of a male to have mated, as well as mating success, was random with respect to size in clear water. This suggests that mate size was not the predominant mate choice cue used by females in this more natural experimental setup where additional cues could be used, and where interaction between the sexes was permitted. This result is in accordance to what has been found for male mate choice in this Baltic Sea population (Sundin et al. 2013). It is, however, in contrast to the population on the Swedish west coast, where females as well as males have been shown to prefer larger mates (Berglund et al. 1986a, b; but see Mobley et al. 2013), and fecundity and body size are closely correlated (Berglund et al. 1986a). The difference in the relationship between male length and number of partners in the turbid and clear treatments found here was due to small males having more partners in the clear than in the turbid treatment, again showing that mate size is not important in mate choice in clear water. Previous research has shown that turbidity relaxes sexual selection on mate size (Järvenpää and Lindström 2004; Sundin et al. 2010; Sundin et al. 2016). Since mating with a larger mate may come with fitness benefits to the offspring in this species (Berglund et al. 1986a; Ahnesjö 1992; Mazzi 2004), turbidity may lead to an enhanced reproductive success.

## Conclusion

When mechanisms of sexual selection were limited to mate choice based on visual cues, turbidity hampered sexual selection (Sundin et al. 2010). We have shown that when additional mechanisms, such as mating competition and mate encounter rates, were allowed to influence sexual selection, turbidity could actually increase sexual selection in a population severely affected by turbidity in nature. Given the increase in human disturbance on ecosystems, studying the impact of environmental stressors is not only important to better understand how environmental fluctuations affect evolutionary processes, but also for conservation biology and studies of the resilience of organisms to anthropogenic induced environmental change.

## Electronic supplementary material

ESM 1 (XLS 121 kb)

ESM 2 (XLS 121 kb)
